# Thermal Comfort; or Popular Hints for Preservation against Colds, Coughs, and Consumption

**Published:** 1843-04

**Authors:** 


Art. X.—*Thermal Comfort; or Popular Hints for Preservation against Colds, Coughs, and Consumption.* By Sir George Lefevre, m.d. &c. —*London,* 1843. 8vo, pp. 31.

We are glad to perceive by the date of this pamphlet that Sir George Lefevre has finally established himself in London; and its contents prove that he is willing to let his countrymen benefit by the experience which his long residence on the continent, and especially in St. Petersburg, has enabled him to acquire. The object of this little essay is to show—

(1) that consumption is much less prevalent in Russia than in England; (2) that the cause of this is not to be sought in the peculiarity of the climate, but in the greater care taken by the inhabitants, out-of-doors and in-doors, by impenetrable clothing, impenetrable walls and windows, and everlasting stoves, to eschew cold and maintain bodily heat; and (3) that the people of England might gain the same happy ends by adopting the same means. He most happily exposes the absurdities almost universally witnessed in our houses, among the rich as well as the poor, of allowing our halls, staircases, and bedrooms to be of the temperature of the wintry blast, while our sitting-rooms are kept at summer heat. Judging from this, foreigners might well imagine, that so far from complaining of our *variable* climate, as we *invariably* do, we were so enamoured of it, that we did all we could to imitate it within doors. We have not room to notice at greater length Sir George's pamphlet, but we commend it to the notice of the profession and the public, as containing something that it interests them to know.

